# How complex must shape data be to model *in vivo* forces? Intraspecific level validation of *in silico* jaw strength estimates in a lizard

**DOI:** 10.1242/jeb.251313

**Published:** 2026-02-09

**Authors:** Stephanie C. Woodgate, Ana Pérez-Cembranos, Valentín Pérez-Mellado, Johannes Müller

**Affiliations:** ^1^ Faculty of Life Sciences, Humboldt-Universität zu Berlin, Unter den Linden 6, 10099 Berlin, Germany; ^2^ Museum für Naturkunde, Leibniz-Institut für Evolutions- und Biodiversitätsforschung, Invalidenstraße 43, 10115 Berlin, Germany; ^3^ Departamento de Biología Animal, Universidad de Salamanca, Campus Miguel de Unamuno s/n. 37007 Salamanca, Spain

**Keywords:** Bite force, Mechanical advantage, Finite element analysis, *Podarcis pityusensis*

## Abstract

A major problem in current biomechanical literature is the extent to which *in silico* data can be validated by *in vivo* data across taxonomic scales. Despite frequent incongruence between *in silico* and *in vivo* data gained from precisely the same individual, biologists and palaeontologists continue to publish *in silico* data of single bones intended to represent entire species. Here, we aim to bridge this gap by investigating whether jaw morphology alone can be used to validate biomechanical models on the intraspecific level in a phenotypically diverse lizard, *Podarcis pityusensis*. We tested this by investigating how effectively *in vivo* bite force measurements from eight populations of this species are predicted by biomechanical models. We used alcohol-preserved specimens from each location to generate population-average and male-average morphologies of mandibles and dentaries, from which we calculated mechanical advantage as well as strength estimates from finite element analysis. Overall, we found a general lack of population-level correlation between *in vivo* and *in silico* data; however, strength estimates from finite element analysis did follow the same bite∼size relationship as *in vivo* bite, suggesting that biomechanical analysis of even a single bone can produce useful bite force estimates. We encourage researchers to create *in silico* models with maximally complex shape data and caution that intraspecific variation is a crucial aspect of *in vivo* and *in silico* biomechanics.

## Teaser

**Summary:** Strength estimates gained from finite element analysis performed on average jaw morphologies of phenotypically diverse *Podarcis pityusensis* populations can accurately reflect *in vivo* bite force∼size relationships.

## INTRODUCTION

The evolution of shape is underpinned by the way biological structures develop and function within the context of their ecological surroundings (Seilacher, 1991; Briggs, 2017). The shape and biomechanical properties of structures can therefore convey a great deal of information about an organism's life. One particularly useful metric to study in terms of vertebrate function and life history is bite force, owing to the roles biting plays in feeding, combat, predator defence and sexual reproduction; further, bite force has been directly linked to reproductive success in some taxa (Lappin and Husak, 2005; Anderson et al., 2008). Studying bite force can therefore shed much light on an organism's life history; however, in many taxa, e.g. extinct species, it is not possible to directly measure bite force. In these cases, biomechanical modelling based on empirical shapes is used to create estimates of bite force.

Bite force estimates gained from computational biomechanical modelling are called ‘*in silico*’ data*.* Two of the most common methods of biomechanically modelling bite force are mechanical advantage (MA) and finite element analysis (FEA). MA is a simple estimator of muscle force transferral, describing efficiency of force transfer by modelling the jaw as a lever with applied and output forces; in general, jaws adapted to higher bite forces should have higher MA (Greaves, 2012; Dumont et al., 2014; Anderson et al., 2008; Throckmorton and Dean, 1994). In FEA, a model of the jaw is divided into a mesh of many small parts, each of which is called an ‘element’ connected to one another by ‘nodes’. Experimental loads simulating muscle forces or forces imposed by food items are then applied to the mesh. The deformation of each element in response to the experimental load is used to calculate metrics such as stress (force per unit area) and strain (deformation of the structure). From stress and strain, estimates of jaw strength can be calculated, a ‘stronger’ jaw indicating the ability to withstand a higher bite force (see Rayfield, 2007 and references within).

FEA has become hugely popular in the field of palaeontology over the last 30 years, allowing jaw strength estimates to be gained directly from fossils (see Rayfield, 2007; Bright, 2014; Mitchell et al., 2025 and references within). Muscle forces are usually estimated by inspection of muscle attachment sites on the fossil, and from muscle force data of the closest available extant relatives (Attard et al., 2016; Button et al., 2014; Varela et al., 2023; Witmer, 1995). Yet, as Bright (2014) highlights, this has the potential to introduce inaccuracies, as the jaw may be used very differently by extant relatives. Further, even in neontological datasets, when bite force data can be gained directly from individuals *in vivo*, the extent to which *in silico* data can be validated by empirical data is unclear.

Validation of *in silico* data can be done via either *in vivo* or *ex vivo* validation. In *ex vivo* validation, strain gauges attached to bone of cadaveric individuals are used to measure bone strain while loads of varying sizes are applied, an approach which allows the same individuals to be used for *ex vivo* analysis and creation of the *in silico* mesh (Bright and Rayfield, 2011; Rayfield, 2011; Toro-Ibacache et al., 2016). Similar approaches may also be used for *in vivo* validation by surgically attaching strain gauges to skull bones to measure strain during biting; the individual may then be euthanized to create a mesh for mechanical analysis (Porro et al., 2013; Panagiotopoulou et al., 2017; Dutel et al., 2021), or different individuals from the same species may be used for creation of models upon which *in silico* modelling will take place (Metzger et al., 2005; Ross et al., 2005, 2011).

To perform *in vivo* bite force validation, individuals are induced to bite on plates, then euthanized for scanning and measurement to create meshes for biomechanical analysis, often with precise muscle force inputs gained from dissection (Gröning et al., 2013; Santana et al., 2010; Davis et al., 2010; Ginot and Blanke, 2024; Ginot et al., 2018; Mara et al., 2010; Kupczik et al., 2007; Püffel et al., 2023). Alternatively, as with strain estimates, different individuals from the same species may be used to create meshes on which *in silico* biomechanical modelling of bite force is performed (Sellers et al., 2017; Laird et al., 2024; Harrison et al., 2025; Huber and Motta, 2004; Curtis et al., 2010; de Zee et al., 2007; Senawi et al., 2015; Becerra et al., 2014).

The extent to which validation is achieved in the above-mentioned studies reflects the often-challenging nature of *in silico* data. Some studies show good agreement between *in vivo* and *in silico* forces when data are sourced from the same individuals, as seen in primates (Panagiotopoulou et al., 2017; Kupczik et al., 2007), murids (Ginot and Blanke, 2024; Ginot et al., 2018), lizards (Gröning et al., 2013) and arthropods (Püffel et al., 2023), or even when different individuals are used to create models, as seen in parrots (Harrison et al., 2025), primates (Ross et al., 2011) and dogfish (Huber and Motta, 2004).

Yet, other studies show incongruence between *in vivo* and *in silico* forces even when data are based on the same individuals, with *in silico* under- or over-estimation of forces, such as in alligators (Porro et al., 2013) and sharks (Mara et al., 2010), as well as when different individuals of the same species are used, such as in lizards (Curtis et al., 2010), primates (Ross et al., 2005) and caviomorph rodents (Becerra et al., 2014). Further studies find more equivocal results, e.g. that *in silico* methods are useful in predicting *in vivo* bites only for certain size ranges, as observed in primates (Laird et al., 2024), bats (Santana et al., 2010; Davis et al., 2010; Senawi et al., 2015) and alligators (Sellers et al., 2017).

The drive to validate *in silico* models over the last 25 years has therefore seen varying levels of success, with studies reporting conflicting ability to reconcile *in vivo* and *in silico* data. This leaves a mismatch in the way that FEA is employed in palaeontological compared with neontological studies, with neontological datasets frequently reporting that validation of *in silico* data cannot be achieved or is only achieved under very specific circumstances. Although the ‘comparative FEA’ approach employed by most palaeontological studies (see Rayfield, 2007; Bright, 2014 and references within), in which jaw strength is compared within the sample, does remove the desire to find precise bite forces from *in silico* analyses, the extent to which this comparative approach can be used across taxonomic scales remains unclear. Indeed, a major problem with current attempts at validation is the differing sources of data used, with studies attempting to validate *in silico* models based on either high-precision *in vivo* data from the very same individuals or *in vivo* data from entirely different individuals of the species, ignoring potential individual and intraspecific variation. In this way, depending on the literature consulted, one could either conclude that bite force is highly individual or is standard across an entire species. It thus remains unclear whether it is valid to estimate jaw strength of a species based on a single bone or single individual.

We argue that a major missing gap in the literature is validation of FEA models at the population level. Taverne et al. (2023) performed FEA on two individuals of *Podarcis siculus* from different populations, finding that the population with the stronger *in vivo* bite force also produced higher *in silico* jaw strength. Although a compelling result, this sample size of two leaves many questions unanswered; particularly, what is the trend across multiple diverse populations? We believe that attempting to validate *in silico* values with *in vivo* data from different populations of the same species remains a major gap in the literature, the bridging of which will allow us to assess to what extent differences in bite force can be detected due to relatively minor differences in morphology (such as those observed at the intraspecific level).

This work attempts to validate *in silico* jaw strength estimates based on biomechanical analysis of alcohol-preserved specimens from eight populations of *Podarcis pityusensis*, the Ibiza wall lizard, with *in vivo* bite force taken during fieldwork at the very same locations. These eight populations originate from different islets, so we can be confident that they are isolated. The species has a fairly conserved head morphology and a highly diverging bite force and diet (Woodgate et al., 2025), making it the perfect candidate for testing validation of *in silico* models at the intraspecific level. This work operated under five hypotheses, using increasingly complex input data for *in silico* methods to estimate jaw strength, comparing each with *in vivo* bite force. We expressly tested whether validation is achievable using the types of data available in natural history collections; namely, bone morphology and estimated muscle forces based on inspection of muscle attachment sites and data from a close phylogenetic relative. We chose not to use population-specific muscle data because these are usually not available in natural history collections, whether degraded during the process of fossilisation, or rendered unreliable due to shrinkage in alcohol-preserved specimens.

Hypothesis 1a is that shrinkage of musculature and body tissues has occurred in the alcohol-preserved lizard dataset. Shrinkage is a known phenomenon within preserved lizards (Vervust et al., 2009) and can limit the extent to which direct measurement of muscle metrics (mass, pennation and length of fibres) are informative inputs into *in silico* models. Further, under hypothesis 1b we predict that lizards that have been preserved in alcohol for a longer time will show greater shrinkage.

Further hypotheses investigate to what extent *in silico* analyses on models generated from warped population-average jaw morphologies can be validated by *in vivo* data, and how complex these models must be for validation to be achieved. Hypothesis 2 is that strength estimates from FEA of average morphologies of a single bone, the dentary, with no estimated muscle forces (as the dentary has no muscle attachment points) will accurately predict *in vivo* bite force. Hypothesis 3 is that MA calculations of population-average morphologies of alcohol-preserved mandibles will accurately predict *in vivo* bite force. Hypothesis 4 is that strength estimates from FEA of average mandible morphologies of alcohol specimens with experimentally applied muscle forces will accurately predict *in vivo* bite force. In all cases, we first tested whether *in silico* bite strength estimates directly correlate with population average bite force, and secondarily tested whether *in silico* and *in vivo* data follow the same allometric relationship.

Finally, hypothesis 5 is that due to the large extent of sexual dimorphism in bite force and body size in this species (Woodgate et al., 2025), male *in silico* jaw strength estimates will show a stronger association with male *in vivo* bite force than total *in silico* jaw strength estimates do with total *in vivo* bite force.

We therefore sought to build a system in which we have empirical links between bite force and shape both *in vivo* and *in silico* at the intraspecific level. We used this system to answer questions surrounding how much input data is needed to create biologically informative biomechanical models.

## MATERIALS AND METHODS

### Study species and location

*Podarcis pityusensis* (Boscá 1883) is a phenotypically diverse lizard species endemic to the Pityusic Islands [comprising Ibiza (Eivissa), Formentera, and the small islets surrounding them] in the Western Mediterranean. Woodgate et al. (2025) reported remarkable diversity in bite force and ecology throughout the natural range of this species. Data were collected from two different sources: lizards in the field and alcohol-preserved specimens; from now on these two sources will be referred to as the ‘method’ of data collection.

### Fieldwork dataset

Bite force and morphology data were collected in the field, reported in Woodgate et al. (2025), under permit ‘Autorizació Especiel per a Capture Científica d'Especies Protegides’, reference CEP 09/2023, issued by Conselleria de Medi Ambient, Govern Balear, for Ana Pérez-Cembranos and Valentín Pérez-Mellado. Bite force was measured based on methods and devices set out in Herrel et al. (1999, 2001). Morphological measurements – snout–vent length (SVL), head height (HH), pileus length (PL), pileus width (PW) and mouth width (MW) – were taken with a steel rule or digital calliper to the nearest 0.01 mm. The dataset used here comprised 229 lizards from eight populations. All measurements are listed in Dataset 1.

### Alcohol dataset

A total of 75 lizards preserved in alcohol were used for *in silico* analysis, representing eight populations of *P. pityusensis*. Specimens are stored in collections as follows: specimens of Bleda Plana, Espartar, Es Vedrà and Trocadors at the University of Salamanca (USAL); specimens from Conillera at the Museum für Naturkunde Berlin (ZMB); specimens from San Antonio at Senckenberg Museum Frankfurt (SMF); and specimens from Espardell and Penjats at Museum Koenig Bonn (ZFMK). Specimens from San Antonio are referred to as ‘Es Pouàs’ from now on, as this collection is the closest available sample of alcohol-preserved lizards to the fieldwork sampling location Es Pouàs. Specimens were identified as male or female either by the dimensions of the head or by femoral pore development, and were also measured for SVL, HH, PL, PW and MW with a digital calliper to the nearest 0.01 mm. Specimens were measured with the same methods as those used in the field. All specimens and measurements are listed in Dataset 2.

### Linear morphometric analysis

All morphometric and statistical analysis was performed in R version 4.5.0 (https://www.r-project.org/). Functions mentioned below are core R functions or part of the package *geomorph* version 4.0.10 (https://CRAN.R-project.org/package=geomorph) unless otherwise specified, and the R script is provided on Zenodo (doi:10.5281/zenodo.17643025). Linear morphometric analysis was used to test whether shape differs between alcohol and fieldwork populations. This was done via a principal components analysis (PCA) based on the morphometric measurements SVL, HH, PL, MW and PW using the function prcomp() in which data were scaled and centred. Data were visualised using the function ggbiplot() from the package *ggbiplot* version 0.6.2 (https://CRAN.R-project.org/package=ggbiplot), which shows the contributions of each morphological metric to each PC axis.

A MANOVA was performed to test associations between linear morphology and location, method, and the interaction between location and method using the function manova() from the package *stats*. The distance in linear morphospace between the fieldwork and alcohol dataset for each population was tested by fitting a linear model with the function lm.rrpp() from the package RRPP version 2.1.2 (https://CRAN.R-project.org/package=RRPP) with 999 iterations on PCA scores according to method and location, then using the pairwise() function with test.type=“dist” and confidence=0.95 to test the significance of the difference.

Duration of alcohol preservation in relation to shape differences was then tested with Spearman correlations using the function cor.test(). The correlation between age (time since collection; see Dataset 2) and distance in morphospace, the percentage difference in SVL, HH, PL and MW between average alcohol-preserved and fieldwork individuals at each location, was tested. In the case of the Es Pouàs specimens, two specimens were collected in 1931 and three were collected in 1957, so the age was averaged as 81 years.

### Geometric morphometric analysis

#### Landmark data

Alcohol-preserved specimens were scanned at the Museum für Naturkunde Berlin using micro-computed tomography (RRID:SCR_022585) with Phoenix nanotom X-ray tubes (Waygate Technologies, Wunstorf, Germany; RRID:SCR_022582) and datos×2.2 software (Waygate Technologies) for cone beam reconstruction. Scans were processed in VG Studio MAX version 3.5 (Volume Graphics GmbH, Heidelberg, Germany) and exported as stereolithography (STL) files (see doi:10.5281/zenodo.17643025). Landmarks were placed on the left mandible in Checkpoint (www.stratovan.com/products/checkpoint), following two different landmarking regimes.

The first landmarking regime was based on 16 point landmarks covering the entire mandible designed to capture gross morphology as described by mandibular sutures (see Fig. S1 and Table S1). The second landmarking regime was based only on the dentary, which was described by seven point landmarks and four curves defined by high-density semi-landmarks, designed to capture higher-precision morphological data (see Fig. S2 and Table S2). Landmark coordinates of each specimen were then exported from Checkpoint as .nts files and imported into R (see doi:10.5281/zenodo.17643025).

### Performing shape analysis

Geometric morphometrics was performed separately for the mandible and dentary landmarking regimes. For the dentary landmarking regime, downsampling was performed to give 13–16 semi-landmarks per curve (see doi:10.5281/zenodo.17643025), and semi-landmarks were defined as sliding along their respective curves using the function equidistantCurve() with 10 iterations.

Generalised Procrustes analysis was performed using the function gpagen(). A PCA was then performed on generalised Procrustes coordinates to create a morphospace defined by PC axes. The function shape.space() from the package *borealis* version 2022.10.27 (https://github.com/aphanotus/borealis) was used to plot morphospaces with PC axes scaled according to the percentage of variation they summarise.

To test whether shape of the mandible and the dentary are significantly determined by location, anova() from the package *car* (https://CRAN.R-project.org/package=car) was used to perform an ANOVA of a linear model based on Procrustes shape data using the function procD.lm() with 999 iterations. Size (measured via log centroid size) and size:shape interaction according to location were also tested. As this showed that shape of the dentary and mandible are significantly different between locations, warps were generated of the average morphology for each population for *in silico* analyses.

### Generating warps of average morphology for each population

USAL_E32h was selected as the reference mesh for warping as it is the closest specimen to the mean in the dentary morphospace which has all teeth still present (the closest was ON_h: 0.0221 from centre, E32_h: 0.0238). For the dentary dataset, the USAL_E32h mandible mesh was imported into Blender version 4.4 (www.blender.org/) and cut at the posterior point of the dentary. The posterior surface was made solid by adding a plane, using internal Blender functions to ensure the mesh was precise and watertight, following methods described by Herbst (https://github.com/evaherbst/Blender_remeshing_guide; Herbst et al., 2022). The cropped ‘dentary’ of USAL_E32h was then exported from Blender in Stanford Triangle Format, i.e. as a .ply file. For the mandible dataset, the ply mesh of the USAL_E32h mandible was directly used. Reference meshes were imported into R using the read.ply() function. The coordinates of the average shape of each population were defined using the function mshape(), and the USAL_E32h mesh was warped to population averages using the function warpRefMesh(). This was repeated for average male morphologies for each population, giving four ‘jaw’ datasets: population average dentary shape, male population average dentary shape, population average mandible shape and male population average mandible shape. All average shapes were exported from R as .ply files (see doi:10.5281/zenodo.17643025).

### Mechanical advantage

Due to significant shrinkage of tissues in the alcohol dataset, and our express wish to test accuracy of *in silico* modelling using data sources available from natural history collections, muscle forces and insertion sites needed to be estimated from morphology and a close phylogenetic relative. Dissection was performed on a specimen of *Podarcis lilfordi* (the sister species of *P. pityusensis*) that had been frozen after death and thawed prior to examination, and images of muscle origin and insertion sites as well as muscle orientation were made. Information gained from this dissection as well as from previous studies (Gröning et al., 2013; Herrel et al., 1998; Taverne et al., 2023) and inspection of bone morphology in each warped mandible was used to discern muscle attachments and lines of action.

MA of each muscle group was calculated following methods set out by Westneat (2003), in which the jaw is modelled as a lever, with the muscle as a pulling force, the food item on the 10th tooth as a load and the articulation point (jaw hinge) as a fulcrum. The 10th tooth was selected to standardize bite point because it is a little over one-third of the way along the toothrow in USAL_E32h (as bite force varies along the toothrow; Tseng et al., 2011). The in-lever is thus defined as the perpendicular distance between the muscle insertion site and the jaw hinge, and the out-lever is the perpendicular distance between the hinge and the bite point (top of the 10th tooth).

Taverne et al. (2023) found there are four muscle groups that contribute to bite force during jaw closure in *Podarcis* lizards: the external jaw adductors, the pseudotemporalis muscles, the adductor posterior and the pterygoideus muscles. As insertion site of the posterior adductor could not be discerned from the site of the external adductor during dissection, one combined insertion site for these two muscle sites was calculated. The in- and out-lever for each of these muscle groups was measured by taking screenshots in lateral and lingual aspect in Blender, upon which lines of muscle insertion, origin and the articulation point were drawn in Inkscape, before measurement was performed in ImageJ version 1.54g (Schneider et al., 2012) using the Blender scale grid.

Data on percentage of total force contributed by each muscle group during biting were obtained from Taverne et al. (2023) based on dissections of *Podarcis siculus* lizards from Adriatic islet Pod Mrčaru. These data were selected because these lizards have similar bite force to *P. pityusensis*; for a gape angle of 35 deg (similar to those recorded in our *in vivo* bite trials; see Dataset 1), the adductors contribute 48.1% of bite force (summing the 45.7% from the external adductors and the 2.4% from the adductor posterior), the pseudotemporalis 31.6% and pterygoideus muscles 20.4%. The pterygoideus muscles are a special case as they have two different insertion points; for this reason, we modelled the resultant as one-half external, one-half internal (i.e. 10.2% for the internal pterygoideus and 10.2% for the external pterygoideus). MA of each muscle group was thus multiplied by the proportion of bite force it contributes, and these were summed to calculate total jaw MA (see Datasets 3 and 4).

As some papers use only the adductor MA as their value for jaw strength, we repeated the same analyses with MA of the adductor alone, hereafter referred to as ‘adductor MA’.

### Finite element analysis

Preparation of meshes for FEA was done in Blender. To make meshes manifold, the Remesh modifier was used to remesh at 0.01 mm for dentaries and 0.02 mm for mandibles. As SVL significantly impacts bite force in this species (Woodgate et al., 2025), warped meshes were scaled to average population size (Mitchell et al., 2025) defined as the distance between landmarks 1 (most anterior) and 3 (most posterior) in both the dentary and the mandible (Tables S1 and S2). Each warped, scaled mesh was then orientated in Blender so that the most anterior point of the dentary is in the negative *x* direction, the occlusal surfaces in the positive *y* and the parasaggital plane in the positive *z*. Meshes were then exported as STL files.

STLs were imported into FEBio version 2.7.0 (Maas et al., 2012). The STL was first meshed (starting with mesh size 1E–04 mm) and then the entire mesh was assigned material properties of bone, an isotropic elastic material with Young's modulus of 17 GPa and Poisson ratio of 0.3, values commonly used for cortical bone in finite element studies, including those of *Podarcis* lizards (Taverne et al., 2023). This was assigned to the entire mesh, including the teeth, which were included owing to their importance in dissipating stress throughout the dentary. Naturally, the materials making up teeth have very different material properties to cortical bone (Zhang et al., 2014); however, as these are warped average morphologies rather than empirical jaws, it was decided that modelling precise material properties of teeth would be an unnecessary source of potential inaccuracy. Boundary conditions and loading procedures were then applied to generate jaw strength estimates as described in the Supplementary Materials and Methods.

### Statistical analyses

All *in silico* and *in vivo* data were logged prior to statistical analysis. To investigate whether strength estimates gained from *in silico* data correlate with *in vivo* bite force on the population level, Kendall correlations were performed using the function cor.test()*.* Kendall correlations were used here as they are non-parametric (i.e. do not assume normality of data) and can be used with small sample sizes (Kendall, 1938).

To test whether *in silico* analysis accurately estimates *in vivo* bite force for a certain head size, models were fitted with estimated bite force as the dependent variable and size (PL for the fieldwork dataset, dentary length for the dentary dataset and mandible length for the mandible datasets) and method, alongside the interaction between size and method, as the independent variables. This was done using the function lm.rrpp() with 999 permutations, as a Shapiro–Wilk test (fitted using function shapiro.test()) revealed that the bite and size datasets are not normally distributed, violating the assumptions of ANOVA (Table S3). For the mandible dataset, *z*-score standardisation was undertaken (see Supplementary Materials and Methods). *In silico* and *in vivo* data were plotted onto common axes for each dataset and interpreted alongside ANOVA results to determine whether *in silico* and *in vivo* data follow the same bite∼size relationship.

## RESULTS

### Linear morphometrics

**Fig. 1.**
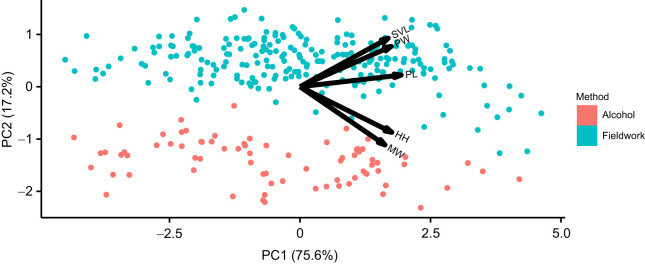
**Linear morphometric analysis results with alcohol-preserved individuals in red, fieldwork individuals in blue.** Black arrows indicate loading of morphological metrics in principal component (PC) axes. *n* for each method: alcohol, 75; fieldwork, 229. SVL, snout–vent length; PW, pileus width; PL, pileus length; HH, head height; MW, mouth width.

Linear PCA analysis returned a morphospace summarised by five principal components. PC1 described 75.6% of shape variation in the sample and PC2 described 17.2% of shape variation (Fig. 1), with further PC axes describing <10% of shape variation (Table S4). PL is the morphology metric which had the greatest contribution to positive PC1 scores, with a loading of 0.494, although all metrics had a loading of over 0.4 (Table S5). MW had the strongest contribution to PC2, with a loading of −0.594, whereas SVL had a positive loading of 0.496. A MANOVA revealed that method, location, and the interaction between method and location determined morphospace occupation (Table 1).

**Table 1.** Summary of results of a MANOVA of linear morphospace occupation according to the method and location

Independent variable	d.f.	Pillai	Approx. *F*	Num. d.f.	Den. d.f.	Pr(>*F*)
Method	1	0.88496	438.49	5	285	<2.2e–16***
Location	7	0.78911	7.74	35	1445	<2.2e–16***
Method:Location	7	0.37001	3.3	35	1445	4.84e–10***
Residuals	289					

Fieldwork and alcohol-preserved populations inhabited a wide range of PC1 but had very little overlap on PC2. Euclidean distances taking into account all PC axes showed that populations differed in the distance in linear morphospace between fieldwork and alcohol-preserved individuals; this distance was significant in all populations apart from Es Pouàs (Table S6), indicating shrinkage. SVL was the morphological metric most different between alcohol-preserved and fieldwork individuals (significant in 5 populations), whereas PL was the least (Table S7). Time since initial preservation showed no correlation with difference in morphology, regardless of whether measured according to morphological metrics or distance in morphospace (Table S8).

### Geometric morphometrics

#### Dentary dataset

**Fig. 2.**
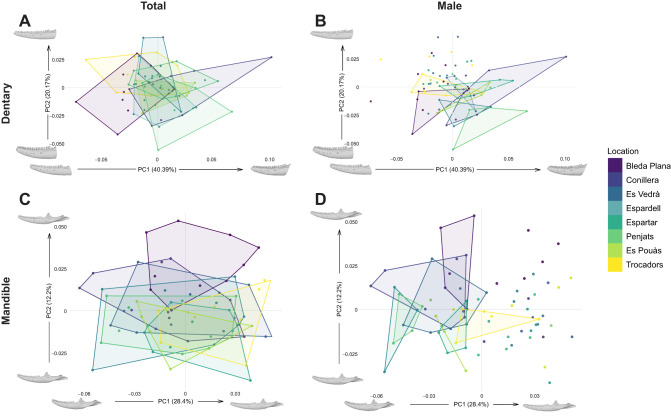
**Geometric morphometric plots.** Grey morphologies represent warped morphologies of minimum and maximum PC values. (A) PC1 and PC2 of the dentary; convex hulls represent total populations. (B) PC1 and PC2 of the dentary; convex hulls represent males by population. (C) PC1 and PC2 of the mandible; convex hulls represent total populations. (D) PC1 and PC2 of the mandible; convex hulls represent males by population. *n* for each location: Bleda Plana, 10; Conillera, 11; Es Pouàs, 5; Es Vedrà, 9; Espardell, 10; Espartar, 9; Penjats, 10; Trocadors, 10. Sample sizes of male hulls: Bleda Plana, 5; Conillera, 8; Es Pouàs, 3; Es Vedrà, 5; Espardell, 3; Espartar, 5; Penjats, 4; Trocadors, 5.

A shape space was generated with 74 total PC axes. PC1 described 40.39% of total shape variation and corresponded to the posterior depth of the dentary, with high PC1 scores indicating a deeper posterior section (see Fig. 2C,D). PC2 described 20.17% of shape variation and corresponded to the elongation of the dentary, with high PC2 scores indicating a narrower, more elongate dentary and low PC2 scores indicating a shorter, deeper dentary. All further PC axes described <10% of shape variation. All locations overlapped in a central section of the space. ANOVAs based on linear models of Procrustes co-ordinates revealed that shape (as well as the interaction between shape and size) was significantly different in different locations (Table S9).

#### Mandible dataset

Geometric morphometrics of the mandible dataset produced a morphospace with 41 total PC axes. PC1 described 28.4% of shape variation and corresponded to depth of the mandible, with high PC1 scores indicating a more elongate, narrow mandible and low PC1 scores indicating a shorter, deeper mandible (see Fig. 2A,B). PC2 described 12.2% of shape variation and corresponded to the curvature of the mandible, with high PC2 scores indicating a more curved surangular crest and low PC2 scores indicating a less curved surangular crest. All other PC axes described <10% of shape variation. ANOVAs based on linear models of Procrustes coordinates revealed that shape (as well as the interaction between shape and size) was significantly different in different locations, although size was not (Table S9).

### Finite element analysis: dentary dataset

**Fig. 3.**
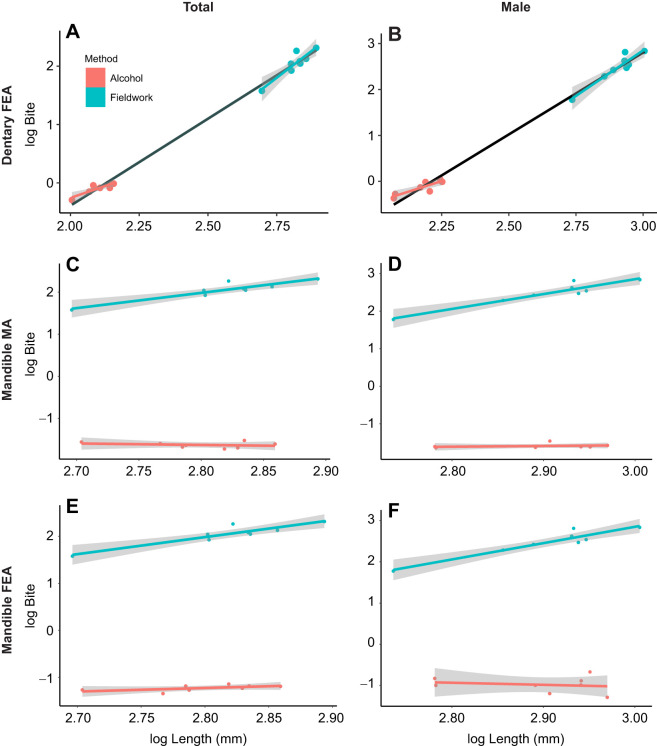
**Plots of the bite∼length relationship of *in vivo* bite force (blue) and *in silico* jaw strength elements (red).** The black line represents both methods, the grey area represents the standard error. Labels indicate how *in silico* jaw strength was estimated. Units for log Bite differ, as described in the Materials and Methods; fieldwork bite force, N; MA jaw strength, dimensionless; FEA jaw strength, 1/(N mm^−2^). Averages are plotted as described in the Materials and Methods, meaning that each of fieldwork and alcohol datasets have *n*=8.

Jaw strength estimates generated from all *in silico* analyses are presented in Table 2. FEA dentary strength estimates and *in vivo* bite force were only marginally correlated at the population level (*P*=0.061; see Table 3). An ANOVA showed that method was not significantly retained in the model linking bite to size, meaning that the slopes of each method were not significantly different to one another (Fig. 3A, Table 4).

**Table 2.** Summary of all results from *in silico* and *in vivo* analysis

Population	*In silico* analysis												*In vivo* analysis			
	Specimens		Mechanical advantage				Finite element analysis strength [1/(N mm^−2^)]						Sample size		Bite force (N)	
			All muscles		Adductor		Dentary		Mandible with muscles		Mandible no muscles					
	Total	Male	Total	Male	Total	Male	Total	Male	Total	Male	Total	Male	Total	Male	Total	Male
Bleda Plana	10	5	0.1779	0.1997	0.2922	0.2659	0.9183	0.9902	0.3182	0.3726	0.3530	0.3759	50	30	8.356	11.68
Conillera	11	8	0.1997	0.2326	0.2713	0.2666	0.9870	0.9849	0.3029	0.3025	0.3029	0.3025	57	29	11.77	17.30
Es Pouàs	5	3	0.2018	0.2006	0.2651	0.2782	0.8604	0.7580	0.2624	0.4366	0.3495	0.3254	21	16	5.277	6.100
Es Vedrà	9	5	0.1825	0.2033	0.2634	0.2730	0.9179	0.8045	0.2905	0.4127	0.3302	0.3488	23	13	9.282	12.17
Espardell	10	3	0.1862	0.1990	0.2577	0.2654	0.9150	1.005	0.3048	0.5117	0.3337	0.4200	21	10	9.008	13.23
Espartar	9	5	0.1943	0.1968	0.2682	0.2689	0.9592	0.8775	0.2804	0.3697	0.3775	0.3676	28	14	11.31	16.93
Penjats	10	4	0.2176	0.2069	0.2687	0.2826	0.9846	1.053	0.3049	0.2761	0.3591	0.4064	19	12	10.09	13.87
Trocadors	10	5	0.2097	0.1945	0.2923	0.2649	0.7461	0.6924	0.2810	0.3694	0.2698	0.2943	10	8	8.615	10.03

Type of analysis employed and dataset (whether total population or males) are labelled. Under ‘*In silico* analysis’, the header ‘Specimens’ refers the number of alcohol-preserved specimens from each population, from which a single population-average morphology was generated for *in silico* analyses.

**Table 3.** Results of Kendall correlations between jaw strength estimates gathered from *in silico* methods (as specified) and *in vivo* bite force

Bite strength estimate method	Equation	*t*	tau	*P*-value
Dentary FEA	Total *in silico*∼*in vivo*	22	0.5714286	0.06101
	Residual total *in silico*∼*in vivo*	15	0.07142857	0.9049
	Male *in silico*∼*in vivo*	20	0.4285714	0.1789
	Residual male *in silico*∼*in vivo*	17	0.2142857	0.5484
Mandible MA	Total∼Bite	15	0.07142857	0.9049
	Residual total *in silico*∼*in vivo*	20	0.4285714	0.1789
	Male *in silico*∼*in vivo*	18	0.2857143	0.3988
	Residual male *in silico*∼*in vivo*	11	−0.2142857	0.5484
Mandible FEA	Total *in silico*∼*in vivo*	0.12468^a^	−0.03636965	0.9008
	Residual total *in silico*∼*in vivo*	9	−0.3571429	0.2751
	Male *in silico*∼*in vivo*	10	−0.2857143	0.3988
	Residual male *in silico*∼*in vivo*	8	−0.4285714	0.1789

FEA, finite element analysis; MA, mechanical advantage.

^a^This is a *z*-value not a *t*-value, as the data included ties.

**Table 4.** Results of ANOVAs testing the association between bite∼size relationships from *in silico* testing of strength via finite element analysis (FEA) of the dentary and *in vivo* bite force

Dentary FEA	Variable	d.f.	SS	MS	*R* ^2^	*F*	*z*	Pr(>*F*)
Bite (total dataset)∼	Length	1	18.6995	18.6995	0.9941	3197.0335	8.1479	0.001***
	Method	1	0.0016	0.0016	0.00009	0.2783	−0.2409	0.598
	Length:Method	1	0.0392	0.0392	0.00208	6.7033	1.882	0.029*
	Residuals	12	0.0702	0.0058	0.00373			
	Total	15	18.8106					
	Final	3	18.74038		0.9962687	1068.005	11.93321	0.001***
Bite (male dataset)∼	Length	1	27.6116	27.6116	0.99157	2788.9875	8.7887	0.001***
	Method	1	0.0259	0.0259	0.00093	2.6201	1.1358	0.123
	Length:Method	1	0.09	0.09	0.00323	9.0911	2.1807	0.011*
	Residuals	12	0.1188	0.0099	0.00427			
	Total	15	27.8463					
	Final	3	27.72753		0.9957336	933.5662	12.68951	0.001***

FEA dentary strength estimates and *in vivo* bite force at the population level were not correlated in males (see Table 3). However, an ANOVA showed that method was not significantly retained in the model linking male bite to size, meaning that the slopes of each method were not significantly different to one another (Fig. 3B, Table 4).

### Mechanical advantage

MA of alcohol-preserved specimens and *in vivo* bite force were not correlated at the population level (see Table 3). An ANOVA showed that method was significantly retained in the model linking bite to size (Fig. 3C, Table 5), meaning that each method followed a bite∼size equation with a significantly different slope. Once bite forces were *z*-score standardised, method was no longer significantly retained; however, a significant model linking bite to size could not be produced, although the model was marginally significant (*P*=0.067).

**Table 5.** Results of ANOVAs testing the association between bite∼size relationships from mechanical advantage (MA) calculations and *in vivo* bite force

Mandible MA	Variable	d.f.	SS	MS	*R* ^2^	*F*	*z*	Pr(>*F*)
Bite (total dataset)∼	Length	1	3.3	3.3	0.06054	446.979	7.2425	0.001***
	Method	1	50.971	50.971	0.93505	6904.137	10.3045	0.001***
	Length:Method	1	0.152	0.152	0.00279	20.628	2.9447	0.002**
	Residuals	12	0.089	0.007	0.00163			
	Total	15	54.511					
	Final	3	54.4229		0.9983748	2457.248	11.58811	0.001***
Bite (*z*-scores total dataset)∼	Length	1	1.9941	1.9941	0.14243	3.1107	1.23821	0.104
	Method	1	0.0785	0.0785	0.00561	0.1225	−0.58467	0.715
	Length:Method	1	4.2349	4.2349	0.30249	6.6063	1.95658	0.021*
	Residuals	12	7.6925	0.641	0.54946			
	Total	15	14					
	Final	3	6.307509		0.4505363	3.279826	1.518402	0.067
Bite (male dataset)∼	Length	1	1.296	1.296	0.01939	148.81	5.5035	0.001***
	Method	1	65.14	65.14	0.9746	7478.146	8.0247	0.001***
	Length:Method	1	0.297	0.297	0.00444	34.092	3.515	0.001***
	Residuals	12	0.105	0.009	0.00156			
	Total	15	66.838					
	Final	3	66.73365		0.9984361	2553.683	13.76338	0.001***
Bite (*z*-scores male dataset)∼	Length	1	5.2524	5.2524	0.37517	8.6559	2.0261	0.018*
	Method	1	0.0194	0.0194	0.00138	0.0319	−1.34677	0.899
	Length:Method	1	1.4466	1.4466	0.10333	2.384	0.98221	0.163
	Residuals	12	7.2816	0.6068	0.52011			
	Total	15	14					
	Final	3	6.718414		0.4798867	3.690632	1.563009	0.055

For males within populations, there was also no correlation between MA of alcohol-preserved populations and *in vivo* bite force (see Table 3). ANOVAs showed the same results as for the total dataset: method was significantly retained in the model linking male bite to size, and once bite forces were *z*-score standardised, method was no longer significantly retained. However, a significant model linking bite to size could not be produced (Table 5), although the final model was marginally significant (*P*=0.055).

### Adductor MA

Adductor MA showed similar trends, with no correlation between populations in adductor MA and *in vivo* bite force (Table S10); however, similar to total MA measurements above, when *z*-score standardised, method was no longer significantly retained (Table S11), with a significant final equation for the total dataset (*P*=0.043), and a marginally significant final equation for the male dataset (*P*=0.066).

### Finite element analysis

#### Mandible dataset

Populations showed no correlation between FEA mandible strength estimates and *in vivo* bite force (see Table 3). An ANOVA showed that method was significantly retained in the model linking bite to size (Fig. 2E, Table 6), meaning that each method followed an equation with a significantly different slope. Once bites were *z*-score standardised, method was no longer significantly retained, meaning slopes were not significantly different.

**Table 6.** Results of ANOVAs testing the association between bite∼size relationships from *in silico* testing of strength via finite element analysis (FEA) of the mandible and *in vivo* bite force

Mandible FEA		d.f.	SS	MS	*R* ^2^	*F*	*z*	Pr(>*F*)
Bite (total dataset)∼	Length	1	3.064	3.064	0.07068	480.522	7.4594	0.001***
	Method	1	40.135	40.135	0.92572	6293.477	10.6771	0.001***
	Length:Method	1	0.079	0.079	0.00183	12.457	2.4212	0.006**
	Residuals	12	0.077	0.006	0.00177			
	Total	15	43.355					
	Final	3	43.27867		0.9982349	2262.152	11.72542	0.001***
Bite (*z*-scores total dataset)∼	Length	1	7.7034	7.7034	0.55024	15.8832	2.74965	0.001***
	Method	1	0.3034	0.3034	0.02167	0.6255	0.19244	0.434
	Length:Method	1	0.1733	0.1733	0.01238	0.3572	−0.12983	0.567
	Residuals	12	5.82	0.485	0.41571			
	Total	15	14					
	Final	3	8.179997		0.5842855	5.621987	2.305464	0.007**
Bite (male dataset)∼	Length	1	0.945	0.945	0.01939	33.062	3.5766	0.001***
	Method	1	47.056	47.056	0.96504	1645.59	6.8438	0.001***
	Length:Method	1	0.416	0.416	0.00854	14.559	2.5848	0.004**
	Residuals	12	0.343	0.029	0.00704			
	Total	15	48.761					
	Final	3	48.41771		0.9929627	564.4034	10.92695	0.001***
Bite (*z*-scores male dataset)∼	Length	1	2.2424	2.2424	0.16017	3.5966	1.3761	0.08
	Method	1	0.0083	0.0083	0.00059	0.0133	−1.4109	0.905
	Length:Method	1	4.2675	4.2675	0.30482	6.8447	1.8712	0.028*
	Residuals	12	7.4818	0.6235	0.53441			
	Total	15	14					
	Final	3	6.518233		0.4655881	3.484863	1.609988	0.062

For males, populations showed no correlation between FEA mandible strength estimates and *in vivo* bite force (see Table 3). An ANOVA showed that method was significantly retained in the model linking male bite to size (Fig. 3, Table 6), meaning that each method followed an equation with a significantly different slope. When bites were *z*-score standardised, method was not significantly retained, but a significant model linking bite to size could not be produced, with a marginally significant *P*=0.062.

#### Mandible with no muscles

The mandible data without muscles produced similar results to that including muscle, suggesting that the estimations of muscle insertion and contribution do not add unnecessary inaccuracy. Populations showed no correlation between FEA strength estimates of mandibles without estimated muscles and *in vivo* bite force, whether including the total dataset or only males (Table S12). Both the total and male datasets significantly retained method in an ANOVA linking bite to size; however, once *z*-score standardisation of bite force took place, method was not retained in either dataset (Table S13).

## DISCUSSION

Many would argue that the only true way to know the bite force of an individual is to measure it *in vivo*; the amount of force an individual can generate is based on integration of the biting structure into the body as a whole. Yet even *in vivo* bite forces may not be enough; there is evidence that bite force varies seasonally in *Pristidactylus* and *Aspidoscelis* lizards (Gowan et al., 2010; Naretto et al., 2025) and behaviourally in humans (Khan et al., 2020). To gain *in vivo* bite data, an individual is stressed, and the stress induces a bite onto bite plates; for this reason, *in vivo* bite forces always have a ‘body condition’ and ‘behavioural’ aspect specific to the exact biting situation. We wish to acknowledge that both the *in vivo* and *in silico* datasets will incorporate some extent of ‘variability’ or ‘error’. In fact, we view this ‘error’ as an intrinsic part of the biological question we are asking; given all the factors that affect biting, can biomechanical models based on average shapes accurately predict average bite forces, and how much input data is needed to make these models informative?

To answer this, we must first check that the correct population-average shapes are being used. All fieldwork populations plot in a significantly different area of linear morphospace compared with their alcohol-preserved counterparts, except Es Pouàs, likely only an exception because of its small alcohol-preserved specimen sample size (5 individuals). SVL has undergone significant shrinkage in five populations, meaning we accept hypothesis 1a with the caveat that shrinkage is apparently not seen in all populations. The extent of shrinkage does not depend on the time since collection, leading us to reject hypothesis 1b. However, this is likely because most notable changes due to shrinkage take place within the first 30 days of preservation (Vervust et al., 2009) and all specimens included in this analysis are at least 40 years old. For this reason, it would not be appropriate to directly measure muscles dissected from alcohol-preserved specimens to inform the loading regime of FEA models. Shrinkage owing to alcohol preservation is well known in a diverse range of taxa; however, studies documenting extent of shrinkage are relatively few considering how much of a problem this phenomenon can pose for morphologists wishing to study alcohol-preserved specimens (Nadeau et al., 2009; Shields and Carlson, 1996; Shu et al., 2017; Reed, 2001; Vervust et al., 2009).

Conversely, in the populations for which head shape metrics are significantly different between alcohol-preserved and fieldwork datasets, alcohol-preserved lizards have larger heads. This may suggest collection preference for individuals with larger heads; however, in this case, it would be surprising that the preference was not also for individuals with greater body size. We suggest instead that phenotypic adaptation has taken place in the time since collection, i.e. in the direction of smaller heads, and that this adaptation has taken place to different extents in different populations. *Podarcis* lizards introduced to islands have been known to evolve significantly different head proportions compared with their source populations within just 30 years (Donihue et al., 2023; Taverne et al., 2023; Herrel et al., 2008).

The lack of population-level correlation between *in silico* and *in vivo* bite force estimates may suggest that jaw morphology is a poor predictor of bite force, and perhaps that ‘comparative FEA’ approaches are not valid at the intraspecific scale. However, we would argue, on the basis that linear morphometrics reports significant changes to morphology and that some methods accurately model the bite∼size relationship in this species, that this finding is an indication that rapid evolution in bite force (as well as body proportions) has taken place since the time of collection. We believe it is thus more useful and biologically informative to focus on the question of whether *in silico* approaches can predict bite force for a given morphology and size rather than for a given population, as we suggest that phenotypes have shifted since collection.

In all cases, the *in silico* data we generate are ‘strength estimates’, different in nature to *in vivo* bite force. The unit of *in vivo* bite force is N, whereas the unit of *in silico* jaw strength is dimensionless for MA and 1/(N mm^−2^) for FEA. These different data types mean that results are less intuitive to compare with one another, meaning that without direct assessment of *in vivo* bone strain, it is impossible to know whether the *in silico* analysis would suggest jaws are able to withstand forces greater or lesser than those experienced *in vivo*, as the units are completely different. However, this is not the question we are seeking to ask; these different data types do still allow for comparison of the bite∼size relationship between *in silico* and *in vivo* data, as they are compared within their own datasets.

MA is the least useful method in terms of estimating the *in vivo* bite∼size relationship from *in silico* data, leading us to reject hypothesis 3. Although the *z*-score standardised adductor MA of the entire dataset does follow the same relationship to size as *in vivo* bite force, all other methods of MA calculation do not show a significant association with *in vivo* data. In contrast, strength estimates gained from FEA of the dentary (for both the total and male datasets), or *z*-score standardised strength estimates from the mandible (muscles: total dataset, no muscles: total and male datasets), follow the same relationship with size as *in vivo* bite, leading us to accept hypotheses 2 and 4.

Our MA calculations may just be too simplistic to model all the ways in which subtle morphological changes can affect force transferal in the jaw; further, our estimates of jaw muscle attachment sites may not be accurate enough. Therefore, we were not able to produce estimates of MA that predict *in vivo* bite forces based on the types of data usually available in natural history collections; we did not find evidence that morphology alone is sufficient to usefully estimate intraspecific-level variation in MA.

The fact that FEA of the dentary is a better predictor of *in vivo* bite force than FEA of the mandible might be considered surprising as it suggests that using a smaller section of the biting structure is more useful than a larger section. However, we suggest this may indicate that higher-density landmarking regimes are more useful at capturing the smaller-scale differences in phenotype that are expected at the intraspecific level. Indeed, dentary geometric morphometric analysis showed greater distances between populations than mandible geometric morphometric analysis. For this reason, we echo the conclusions of Ginot et al. (2019) and Toro-Ibacache et al. (2016), proposing that maximally complex shape data should be used if aiming to create accurate FEA models.

Having stated this, it may also be considered surprising that the male *in silico* dataset is not better at predicting male *in vivo* bite forces than the total *in silico* dataset is at predicting total *in vivo* bite, owing to the high sexual dimorphism observed in the field (Woodgate et al., 2025). This leads us to reject hypothesis 5. Divergence between populations in *in vivo* male bite force shows significant association with mouth width, which is not the case for female bite force (Woodgate et al., 2025). This suggests that *in vivo* male bites may be more influenced by muscle size, which may have the result that the shape of the mandible alone (and simulation of muscle forces) is less effective at predicting bite force in males.

We could not achieve population-level validation of our *in silico* and *in vivo* bite estimates. We believe this is because our input meshes for *in silico* analysis were not precise enough to their population, i.e. alcohol-preserved specimens had been collected a long enough time ago that populations have shifted in phenotype since collection. Therefore, we conclude by cautioning against use of single individuals as representatives of bite force of entire species; our dataset suggests significant phenotypic change in <100 years despite originating from highly conserved collection points, showing how inadequate individual *in silico* bite force estimates can be as a represention of entire species.

Nevertheless, our *in silico* analyses do follow the same fundamental allometric relationship of bite∼size as our *in vivo* dataset. Phenotypic shifts within populations may take place over relatively short periods of time, but the allometric relationship of bite force is less evolutionarily labile. Therefore, we do show here that *in silico* biomechanical modelling of alcohol-preserved specimens can be used to predict *in vivo* bite estimates, even in cases where phenotypic adaptation has taken place since the time of collection. We therefore encourage that studies using *in silico* methods should focus on inputting maximally complex input shape data when aiming to model strength, and we advise consideration of intraspecific variability, especially when employing a ‘comparative FEA’ framework.

## Supplementary Material

10.1242/jexbio.251313_sup1 Supplementary information

Dataset 1. Fieldwork dataset used in analysis.

Dataset 2. Alcohol-preserved specimen dataset used in analysis.

Dataset 3. Calculation of Mechanical Advantage based on average mandible morphology of the total population.

Dataset 4. Calculation of Mechanical Advantage based on average male mandible morphology.

Dataset 5. Finite Element Analysis data of models based on population average dentary shapes.

Dataset 6. Finite Element Analysis data of models based on average male dentary shapes.

Dataset 7. Finite Element Analysis data of models based on population average mandible shapes, with estimated muscle forces.

Dataset 8. Finite Element Analysis data of models male mandible based on average shapes, with estimated muscle forces.

Dataset 9. Finite Element Analysis data of models based on population average mandible shapes, without muscle forces.

Dataset 10. Finite Element Analysis data of models based on average male mandible shapes, without muscle forces.
