# Supplementary material for: How complex must shape data be to model in vivo forces? Intraspecific level validation of in silico jaw strength estimates in a lizard
Source: J Exp Biol. 2026 Feb 9;229(3):jeb251313. doi: 10.1242/jeb.251313 (PMC12951603; doi:10.1242/jeb.251313)
Supplement: Supplementary information [file jexbio-229-251313-s1.pdf]

**Supplementary Materials and Methods**

Boundary conditions for the FEA analysis:

*Dentary loading procedure.* Nodal boundary conditions of zero displacement in the x, y, z directions were applied at the posterior flat face of the dentary. A linear pressure with symmetrical stiffness of 10 $Nmm^{-2}$ was applied across the entire top of the $10^{th}$ tooth from dorsal view to simulate forces experienced during biting.

*Mandible loading procedure: scaled musculature.* Data from the dissection of the *Podarcis lilfordi* specimen, as well as (Gröning et al. 2013; Herrel et al. 1998; Taverne et al. 2023) and bone morphology in each warped mandible were used to discern muscle attachments and lines of action, as described in the Methods. Muscle centroids are specific to the warp but lines of action are not (as only the mandible was warped not the whole skull).

An experimental value of 100N for total muscle force was chosen following methods of Mitchell et al. (2025). Following data reported by Taverne et al. (2023) as described above, the total muscle force for each mesh was divided up between the four muscle groups involved in *Podarcis* bite force. However, in nature, larger individuals have larger muscles (see (Mitchell et al. 2025; Gröning et al. 2013) for information on the importance of muscle scaling in FEA), so to determine muscle forces, muscle scaling according to mandible volume was performed. The closest mandible to the average volume was the Espartar warp which had a volume of 11.27$mm^2$. Therefore, 100N force was applied to the Espartar mesh, while all other warps had muscle forces calculated as (1):

$$100[(mandible\ volume - \ Espartar\ mandible\ volume)^{2/3}]$$

following methods set out by (Mitchell et al. 2025; Strait et al. 2010). This gave a total muscle force value which was then divided up according to muscle group following data reported by Taverne et al. (2023) as specified above.

As these are resultant forces (acting along their line of action) they must be solved for x- y- and z- components before being applied as loads. Images taken of drawn lines of action in Blender were used to estimate the angle at which the pulling force of the muscle acts to the bone in ImageJ. SOHCAHTOA was used to solve this for x- y- and z- components. Muscle forces were applied as surface traction forces (Grosse et al. 2007) as close to the centroid as possible. Boundary conditions of zero displacement at the jaw hinge and the entire top of the $10^{th}$ tooth (to simulate bite point) were applied.

Journal of Experimental Biology • Supplementary information

*Mandible loading procedure: no muscles.* As specified above, modelling of musculature required assumptions and estimations which may be a source of unnecessary inaccuracy. For this reason, FEA was repeated with mandibles with no muscle forces, modelled in a similar way to the dentary, the posterior constraint kept as the jaw hinge, and a linear pressure force of 10 $Nmm^{-2}$ with symmetrical stiffness was placed on the top of the $10^{th}$ tooth.

Generating jaw strength estimates from FEA analysis

*Calculating jaw strength from FEA.* Effective stress of each element at the final timestep (timestep 10) was exported. The elements undergoing the top 1% of stress values were removed following methods specified by Marcé-Nogué et al. (2017), as these often represent artificial noise near loads or constraints, rather than actual biological signal. Thresholds for how much of the mesh should have values removed to account for artificial noise vary according to publication from 1% (Mitchell et al. 2025) to 5% (Walmsley et al. 2013). The mean effective stress was then calculated.

To assess convergence, analysis was iteratively repeated on meshes of increasing element number, until a mesh was found in which the average effective stress experienced at timestep 10 was <2% different to the mesh tested before (achieved in all cases apart from the Conillera mandible FEA with no applied muscle forces (see Supplementary Data Sheet 9)). Because the models converged at different values of mesh size (see Supplementary Data Sheets 5-10), it is important to control for how element size may affect values of stress in the mesh. This was done by calculating the Mesh-Weighted Arithmetic Mean (MWAM) with unit $Nmm^{-2}$, as described by Marcé Nogué et al. (2016), specified as (2):

$$MWAM = \frac{Sum(Element\ Effective\ Stress \times Element\ volume)}{Total\ volume}$$

The percentage error of the mesh was also investigated; this looks at how homogeneous the mesh is, comparing the difference between the MWAM and the mean effective stress (3):

$$Error = \frac{MWAM - Average\ Effective\ Stress}{MWAM} \times 100$$

A ‘strength estimate’ was then generated with unit 1/ $Nmm^{-2}$ (4):

$$Strength\ estimate = \frac{1}{MWAM}$$

The strength estimate was then used in statistical analyses. All mesh data, including convergence data, can be found in Supplementary Data Sheets 5-10.

Z-score standardisation:

For the mandible dataset, log mandible length (mm) and log pileus length (mm) share the same scale and the same units (values between 2 and 3), but strength estimate (gained either through FEA or through MA calculations) does not share the same units or scale (MA: dimensionless, FEA: 1/Nmm$^{-2}$, *in vivo* bite: N). For this reason, in the cases where initial results showed significant difference in bite force according to method, within-group z-score standardization was performed for bite. The z-score reflects how many standard deviations one data point is away from the mean of the group. The z-score was calculated separately for *in silico* and *in vivo* bites, according to the equation (5):

$$z = \frac{x - \bar{x}}{sd}$$

in which $\bar{x}$ is the group mean and $sd$ is the group standard deviation (Kreyzig 1979). The same procedure of statistical analysis as described in the Methods was then repeated with the z-score bite data.

**Table S1.** Landmarks used for geometric morphometric analysis of the mandible.

| **Landmark name** | **Landmark description** |
|---|---|
| Most anterior | Front tip of the mandible |
| Coronoid top | Most ventral tip of the coronoid |
| Mandible posterior | Posterior tip of the mandible |
| Angular anterior | The most anterior point of the angular |
| Surangular anterior | The most anterior point of the surangular visible in the lateral aspect |
| Anterior coronoid | The most anterior point of the coronoid |
| Coronoid dentary angular | Point at which the coronoid, dentary and angular meet |
| Coronoid posterior | The most posterior point of the coronoid |
| Surangular angular dentary | Point at which the surangular, dentary and angular meet |
| Dentary posterior | Posterior point of the dentary |
| Angular posterior | Posterior point of the angular |
| Reticular low | Lowest point of the reticular |
| Surangular interior | Most interior point of the coronoid articulation |
| Surangular exterior | Most exterior point of the coronoid articulation |
| Toothrow coronoid | Point at which the toothrow and coronoid meet |
| Dentary coronoid | Point on the lingual surface at which the dentary and coronoid meet |

**Table S2.** Landmarks used for geometric morphometric analysis of the dentary.

| **Landmark name** | **Landmark description** |
|---|---|
| Most anterior | Front tip of the mandible |
| Highest posterior | Posterior highest point of the dentary |
| Lowest posterior | Posterior lowest point of the dentary |
| Anterior tooth | Front surface of the front tooth |
| Posterior tooth | Back surface of the back tooth |
| Toothrow posterior | Posterior point of the toothrow |
| Toothrow anterior | Anterior point of the toothrow |
| Ventral surface | Curve defined by 58 semi-landmarks (downsampled to 14 semi-landmarks in R) sliding over the ventral surface between the most anterior and the highest posterior |
| Dorsal surface | Curve defined by 61 semi-landmarks (downsampled to 14 semi-landmarks in R) sliding over the dorsal surface between the most anterior and the lowest posterior |
| Ventral toothrow | Curve defined by 69 semi-landmarks (downsampled to 16 semi-landmarks in R) sliding over the ventral surface of the toothrow between the toothrow anterior and toothrow posterior |
| Dorsal toothrow | Curve defined by 55 semi-landmarks (downsampled to 13 semi-landmarks in R) sliding over the dorsal surface of the toothrow between the toothrow anterior and toothrow posterior |

**Table S3.** Showing results of tests of normality and homogeneity of variances for a given dataset.

| Metric | Test value | Population average | | Male average | |
|---|---|---|---|---|---|
| | | Response | P-value | Response | P-value |
| Bite (MA + fieldwork) | Shapiro-Wilk (W-value) | 0.7035 | 0.0001845*** | 0.70908 | 0.0002128*** |
| | Levene (F-value): by method | 2.5284 | 0.1341 | 5.6899 | 0.03175* |
| Bite (adductor MA + fieldwork) | Shapiro-Wilk (W-value) | 0.70182 | 0.0001767*** | 0.70855 | 0.0002099*** |
| | Levene (F-value): by method | 4.0119 | 0.06494 | 6.7364 | 0.02116* |
| Bite (dentary FEA + fieldwork) | Shapiro-Wilk (W-value) | 0.75013 | 0.0006375*** | 0.77528 | 0.001303** |
| | Levene (F-value): by method | 2.1637 | 0.1634 | 1.4157 | 0.2539 |
| Bite (mandible FEA + fieldwork) | Shapiro-Wilk (W-value) | 0.7096 | 0.0002157*** | 0.75561 | 0.0007429*** |
| | Levene (F-value): by method | 2.774 | 0.118 | 1.0005 | 0.3342 |
| Bite (mandible FEA no muscles + fieldwork) | Shapiro-Wilk (W-value) | 0.7243 | 0.0003167** | 0.73854 | 0.0004639*** |
| | Levene (F-value): by method | 1.5244 | 0.2373 | 2.7344 | 0.1204 |
| Length (dentary FEA + fieldwork) | Shapiro-Wilk (W-value) | 0.75632 | 0.0007577*** | 0.79301 | 0.002203** |
| | Levene (F-value): by method | 0.0014 | 0.9711 | 0.0172 | 0.8975 |
| Length (mandible FEA + fieldwork) | Shapiro-Wilk (W-value) | 0.92159 | 0.1789 | 0.87864 | 0.03697* |
| | Levene (F-value): by method | 1E-04 | 0.9917 | 0.0154 | 0.9029 |

**Table S4.** Summary of data from PC axes from linear morphometric analysis.

| Metric | PC1 | PC2 | PC3 | PC4 | PC5 |
|---|---|---|---|---|---|
| Standard deviation | 1.9439 | 0.9266 | 0.42029 | 0.31288 | 0.29645 |
| Proportion of Variance | 0.7558 | 0.1717 | 0.03533 | 0.01958 | 0.01758 |
| Cumulative proportion | 0.7558 | 0.9275 | 0.96284 | 0.98242 | 1 |

Journal of Experimental Biology • Supplementary information

**Table S5.** Summary of loadings of each morphology metric on each principal component axis.

| Morphology metric | PC1 | PC2 | PC3 | PC4 | PC5 |
|---|---|---|---|---|---|
| SVL | 0.43042 | 0.49568 | 0.65887 | 0.20243 | 0.30652 |
| PL | 0.49367 | 0.11919 | 0.06127 | -0.3585 | -0.7809 |
| PW | 0.44516 | 0.41166 | -0.7489 | 0.16674 | 0.20895 |
| HH | 0.44812 | -0.4661 | 0.01391 | -0.5896 | 0.48391 |
| MW | 0.4148 | -0.5945 | 0.03212 | 0.6746 | -0.1357 |

**Table S6.** Pairwise comparison of linear according to morphometric data, each group method of data collection.

| Pairwise comparison | d | UCL(-95%) | Z | Pr>d |
|---|---|---|---|---|
| ALCOHOL Bleda Plana : FIELDWORK Bleda Plana | 1.913457 | 1.344153 | 2.515569 | 0.006** |
| ALCOHOL Conillera : FIELDWORK Conillera | 2.398732 | 1.3046234 | 3.023492 | 0.001** |
| ALCOHOL Es Pouàs : FIELDWORK Es Pouàs | 1.962607 | 2.043831 | 1.609437 | 0.065 |
| | | | | |
| ALCOHOL Espardell : FIELDWORK Espardell | 2.226446 | 1.5685815 | 2.524933 | 0.002** |
| ALCOHOL Espartar : FIELDWORK Espartar | 2.084392 | 1.4278951 | 2.489499 | 0.003** |
| ALCOHOL Penjats : FIELDWORK Penjats | 2.160179 | 1.563731 | 2.437089 | 0.003** |
| ALCOHOL Trocadors : FIELDWORK Trocadors | 2.732495 | 1.796412 | 2.65662 | 0.002** |

**Table S7.** Shrinkage according to morphological metric and location. % diff indicates the percentage difference from the fieldwork to the alcohol dataset. P-value indicates the significance of difference between fieldwork and alcohol datasets.

| Population | SVL | | HH | | PL | | MW | |
|---|---|---|---|---|---|---|---|---|
| | % diff | P | % diff | P | % diffe | P | % diff | P |
| Bleda Plana | -8.1397 | 0.74 | 15.82633 | 0.61 | -2.40674 | 1 | 23.83863 | 7.87E-03** |
| Conillera | -21.1291 | 3.20E-10*** | -0.34682 | 1 | -8.24629 | 1 | 4.98008 | 1 |
| Es Pouas | -1.05605 | 1 | 21.8126 | 1 | 4.438467 | 1 | 27.59949 | 0.20789 |
| Es Vedra | -16.6512 | 1.50E-04*** | 8.885942 | 1 | -7.59566 | 1 | 12.48568 | 1 |
| Espardell | -12.768 | 2.37E-02* | 13.52406 | 1 | -8.44496 | 1 | 15.625 | 0.92536 |
| Espartar | -14.0407 | 3.20E-03** | 15.8042 | 0.92 | -6.08387 | 1 | 18.88361 | 0.2 |
| Penjats | -1.64729 | 1 | 32.72251 | 2.90E-05*** | -3.2014 | 1 | 25.49889 | 3.96E-03** |
| Trocadors | -18.0462 | 7.01E-03** | 4.494382 | 1 | -13.3896 | 1 | 14.04358 | 1 |

**Table S8.** Table summarizing the results of Spearman correlations between collection age and percentage difference in morphological metrics and distance in linear morphospace.

| Metric | rho | S | p-value |
|---|---|---|---|
| % difference in SVL | -0.0383 | 87.218 | 0.9282 |
| % difference in HH | 0.02554 | 81.855 | 0.9521 |
| % difference in PL | 0.08939 | 76.491 | 0.8333 |
| % difference in MW | 0.06385 | 78.637 | 0.8806 |
| Morphospace distance | 0.29372 | 59.328 | 0.4801 |

**Table S9.** Summarising results of ANOVAs of Procrustes co-ordinates of alcohol specimens (based on mandible or dentary datasets, as specified), according to size, shape and the interaction of size and shape.

| Dataset | | | Df | SS | MS | Rsq | F | Z | Pr(>F) |
|---|---|---|---|---|---|---|---|---|---|
| Mandible | Csize~Location | Location | 7 | 0.20689 | 0.029556 | 0.12186 | 1.3282 | 0.64992 | 0.265 |
| | | Residuals | 67 | 1.4909 | 0.022252 | 0.87814 | | | |
| | | Total | 74 | 1.69779 | | | | | |
| | Shape~Location | Location | 7 | 0.041911 | 0.005987 | 0.18846 | 2.2227 | 4.1519 | 0.001*** |
| | | Residuals | 67 | 0.180482 | 0.002694 | 0.81154 | | | |
| | | Total | 74 | 0.222393 | | | | | |
| | Csize*Shape~Location | Location | 7 | 0.4103 | 0.058616 | 0.12845 | 1.4107 | 1.9824 | 0.021* |
| | | Residuals | 67 | 2.784 | 0.041552 | 0.87155 | | | |
| | | Total | 74 | 3.1943 | | | | | |
| Dentary | Csize~Location | Location | 7 | 0.12588 | 0.017983 | 0.10106 | 1.0761 | 0.27082 | 0.401 |
| | | Residuals | 67 | 1.11967 | 0.016711 | 0.89894 | | | |
| | | Total | 74 | 1.24555 | | | | | |
| | Shape~Location | Location | 7 | 0.023209 | 0.0033156 | 0.17713 | 2.0603 | 2.9612 | 0.003** |
| | | Residuals | 67 | 0.107819 | 0.0016092 | 0.82287 | | | |
| | | Total | 74 | 0.131029 | | | | | |
| | Csize*Shape~Location | Location | 7 | 0.31384 | 0.044834 | 0.17705 | 2.0591 | 2.9552 | 0.003** |
| | | Residuals | 67 | 1.4588 | 0.021773 | 0.82295 | | | |
| | | Total | 74 | 1.77264 | | | | | |

**Table S10.** Results of of Kendall correlations be from adductor tween 'bite' values gathered MA measurement (as specified) and *in-vivo* bite.

| | | T | tau | p-value |
|---|---|---|---|---|
| ADDUCTOR MA | TOTAL *in silico ~ in vivo* | 14 | 0 | 1 |
| | RESIDUAL TOTAL *in silico ~ in vivo* | 17 | 0.2142857 | 0.5484 |
| | MALE *in silico ~ in vivo* | 15 | 0.07142857 | 0.9049 |
| | RESID MALE *in silico ~ in vivo* | 16 | 0.1428571 | 0.7195 |

Journal of Experimental Biology • Supplementary information

**Table S11.** Results of ANOVAs testing the association between bite ~ size relationships from *in-silico* testing of strength via adductor MA and *in-vivo* bite force.

| | | Df | SS | MS | Rsq | F | Z | Pr(>F) |
|---|---|---|---|---|---|---|---|---|
| Bite (total dataset)~ | LENGTH | 1 | 2.858 | 2.858 | 0.06317 | 487.205 | 7.4641 | 0.001*** |
| | METHOD | 1 | 42.166 | 42.166 | 0.93195 | 7187.244 | 10.3266 | 0.001*** |
| | LENGTH:METHOD | 1 | 0.15 | 0.15 | 0.00332 | 25.611 | 3.0936 | 0.001*** |
| | Residuals | 12 | 0.07 | 0.006 | 0.00156 | | | |
| | Total | 15 | 45.244 | | | | | |
| | Final | 3 | 45.17409 | | 0.998444 | 2566.687 | 11.84552 | 0.001*** |
| Bite (z-scores total dataset)~ | LENGTH | 1 | 1.5899 | 1.5899 | 0.11356 | 2.6027 | 1.17033 | 0.122 |
| | METHOD | 1 | 0.0626 | 0.0626 | 0.00447 | 0.1025 | -0.70165 | 0.742 |
| | LENGTH:METHOD | 1 | 5.0175 | 5.0175 | 0.35839 | 8.2141 | 1.9837 | 0.021* |
| | Residuals | 12 | 7.3301 | 0.6108 | 0.52358 | | | |
| | Total | 15 | 14 | | | | | |
| | Final | 3 | 6.66994 | | 0.4764243 | 3.639774 | 1.714647 | 0.043* |
| Bite (male dataset)~ | LENGTH | 1 | 1.17 | 1.17 | 0.02019 | 160.025 | 5.6715 | 0.001*** |
| | METHOD | 1 | 56.354 | 56.354 | 0.9727 | 7710.67 | 8.3171 | 0.001*** |
| | LENGTH:METHOD | 1 | 0.324 | 0.324 | 0.0056 | 44.398 | 3.7139 | 0.001*** |
| | Residuals | 12 | 0.088 | 0.007 | 0.00151 | | | |
| | Total | 15 | 57.936 | | | | | |
| | Final | 3 | 57.84838 | | 0.9984862 | 2638.364 | 14.11553 | 0.001*** |
| Bite (z-scores male dataset)~ | LENGTH | 1 | 3.8425 | 3.8425 | 0.27446 | 5.9881 | 1.6843 | 0.036* |
| | METHOD | 1 | 0.0142 | 0.0142 | 0.00101 | 0.0221 | -1.2348 | 0.869 |
| | LENGTH:METHOD | 1 | 2.4431 | 2.4431 | 0.1745 | 3.8072 | 1.434 | 0.062 |
| | Residuals | 12 | 7.7003 | 0.6417 | 0.55002 | | | |
| | Total | 15 | 14 | | | | | |
| | Final | 3 | 6.299711 | | 0.4499794 | 3.272455 | 1.490914 | 0.066 |

**Table S12.** Results of of Kendall correlations between strength estimates gathered from mandible FEA without muscles (as specified) and *in-vivo* bite.

| | | T | tau | p-value |
|---|---|---|---|---|
| MANDIBLE FEA NO MUSCLES | TOTAL *in silico ~ in vivo* | 20 | 0.4285714 | 0.1789 |
| | RESIDUAL TOTAL *in silico ~ in vivo* | 16 | 0.1428571 | 0.7195 |
| | MALE *in silico ~ in vivo* | 20 | 0.4285714 | 0.1789 |
| | RESIDUAL MALE *in silico ~ in vivo* | 13 | -0.07142857 | 0.9049 |

Journal of Experimental Biology • Supplementary information

**Table S13.** Results of ANOVAs testing the association between bite ~ size relationships from *in-silico* testing of strength via Finite Element Analysis of the mandible with no muscles and *in-vivo* bite force.

| | | Df | SS | MS | Rsq | F | Z | Pr(>F) |
|---|---|---|---|---|---|---|---|---|
| Bite (total dataset)~ | LENGTH | 1 | 3.126 | 3.126 | 0.07939 | 417.47 | 7.1561 | 0.001*** |
| | METHOD | 1 | 36.122 | 36.122 | 0.91744 | 4824.027 | 9.8558 | 0.001*** |
| | LENGTH:METHOD | 1 | 0.035 | 0.035 | 0.00089 | 4.662 | 1.531 | 0.063 |
| | Residuals | 12 | 0.09 | 0.007 | 0.00228 | | | |
| | Total | 15 | 39.373 | | | | | |
| | Final | 3 | 39.28304 | | 0.9977178 | 1748.72 | 11.707 | 0.001*** |
| Bite (z-scores total dataset)~ | LENGTH | 1 | 9.7084 | 9.7084 | 0.69345 | 29.801 | 3.3047 | 0.002** |
| | METHOD | 1 | 0.3823 | 0.3823 | 0.02731 | 1.1736 | 0.5213 | 0.313 |
| | LENGTH:METHOD | 1 | 0 | 0 | 0 | 0.0001 | -2.2659 | 0.992 |
| | Residuals | 12 | 3.9093 | 0.3258 | 0.27923 | | | |
| | Total | 15 | 14 | | | | | |
| | Final | 3 | 10.0907 | | 0.7207659 | 10.3249 | 3.140143 | 0.002** |
| Bite (male dataset)~ | LENGTH | 1 | 1.502 | 1.502 | 0.0304 | 165.143 | 5.775 | 0.001*** |
| | METHOD | 1 | 47.662 | 47.662 | 0.96456 | 5240.315 | 7.4484 | 0.001*** |
| | LENGTH:METHOD | 1 | 0.14 | 0.14 | 0.00283 | 15.378 | 2.6803 | 0.003** |
| | Residuals | 12 | 0.109 | 0.009 | 0.00221 | | | |
| | Total | 15 | 49.413 | | | | | |
| | Final | 3 | 49.30351 | | 0.9977912 | 1806.945 | 13.89668 | 0.001*** |
| Bite (z-scores male dataset)~ | LENGTH | 1 | 11.4131 | 11.4131 | 0.81522 | 53.8283 | 4.2119 | 0.001*** |
| | METHOD | 1 | 0.0421 | 0.0421 | 0.00301 | 0.1986 | -0.3937 | 0.666 |
| | LENGTH:METHOD | 1 | 0.0005 | 0.0005 | 0.00004 | 0.0023 | -1.8205 | 0.955 |
| | Residuals | 12 | 2.5443 | 0.212 | 0.18174 | | | |
| | Total | 15 | 14 | | | | | |
| | Final | 3 | 11.45567 | | 0.8182623 | 18.00974 | 4.072041 | 0.001*** |

Journal of Experimental Biology • Supplementary information

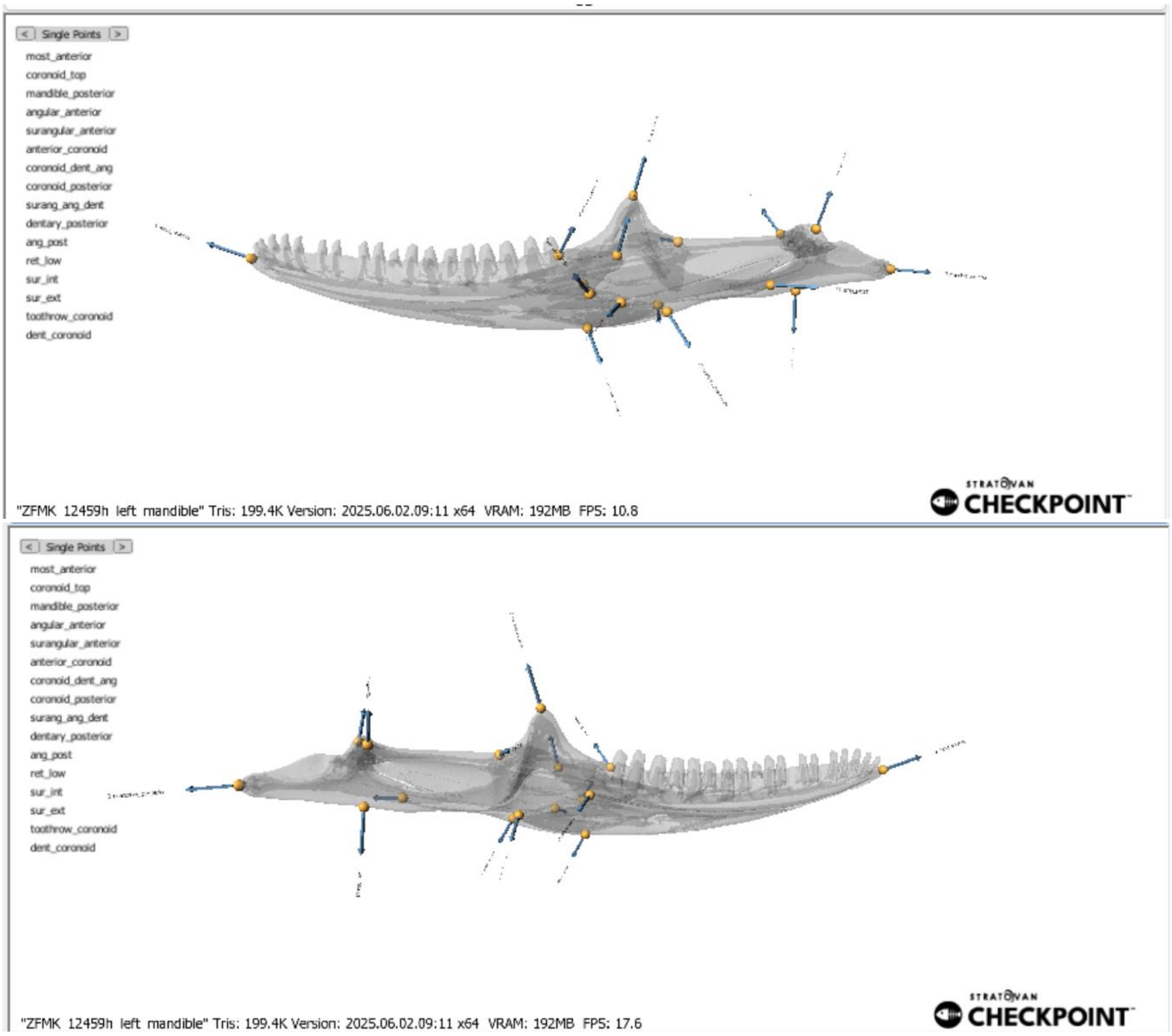


**Fig. S1.** Mandible landmarking regime showing ZFMK_12459h mandible in (A) lateral view and (B) sagittal view.

Journal of Experimental Biology • Supplementary information

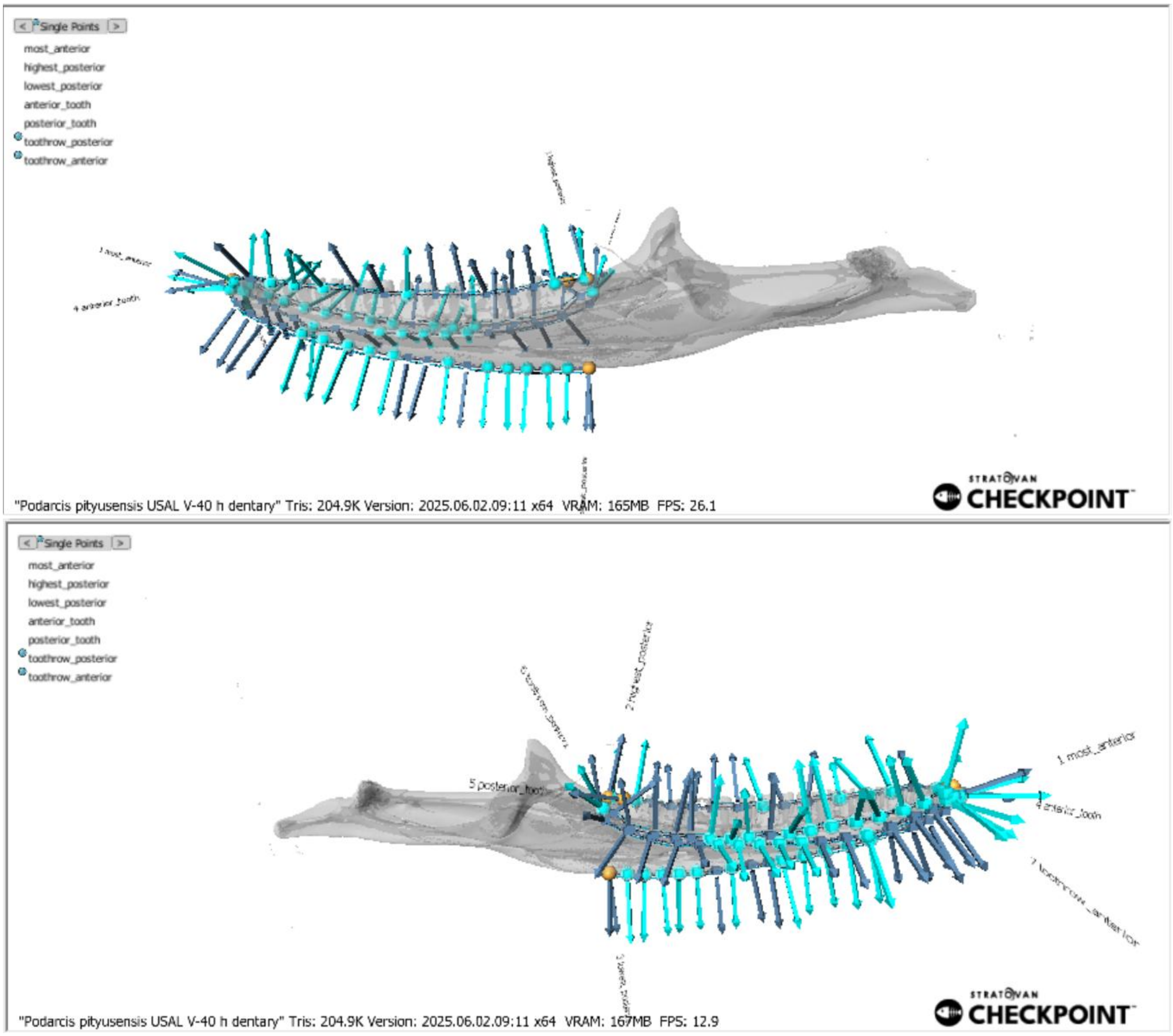


**Fig. S2.** Dentary landmarking regime showing USAL_V_40h mandible in (A) lateral view and (B) sagittal view.

**Dataset 1.**Fieldwork dataset used in analysis.

Available for download at
https://journals.biologists.com/jeb/article-lookup/doi/10.1242/jeb.251313#supplementary-data

**Dataset 2.** Alcohol-preserved specimen dataset used in analysis.

Available for download at
https://journals.biologists.com/jeb/article-lookup/doi/10.1242/jeb.251313#supplementary-data

**Dataset 3.** Calculation of Mechanical Advantage based on average mandible morphology of the total population.

Available for download at
https://journals.biologists.com/jeb/article-lookup/doi/10.1242/jeb.251313#supplementary-data

**Dataset 4.** Calculation of Mechanical Advantage based on average male mandible morphology.

Available for download at
https://journals.biologists.com/jeb/article-lookup/doi/10.1242/jeb.251313#supplementary-data

**Dataset 5.** Finite Element Analysis data of models based on population average dentary shapes.

Available for download at
https://journals.biologists.com/jeb/article-lookup/doi/10.1242/jeb.251313#supplementary-data

**Dataset 6.** Finite Element Analysis data of models based on average male dentary shapes.

Available for download at
https://journals.biologists.com/jeb/article-lookup/doi/10.1242/jeb.251313#supplementary-data

**Dataset 7.** Finite Element Analysis data of models based on population average mandible shapes, with estimated muscle forces.

Available for download at
https://journals.biologists.com/jeb/article-lookup/doi/10.1242/jeb.251313#supplementary-data

**Dataset 8.** Finite Element Analysis data of models male mandible based on average shapes, with estimated muscle forces.

Available for download at
https://journals.biologists.com/jeb/article-lookup/doi/10.1242/jeb.251313#supplementary-data

**Dataset 9.** Finite Element Analysis data of models based on population average mandible shapes, without muscle forces.

Available for download at
https://journals.biologists.com/jeb/article-lookup/doi/10.1242/jeb.251313#supplementary-data

**Dataset 10.** Finite Element Analysis data of models based on average male mandible shapes, without muscle forces.

Available for download at
https://journals.biologists.com/jeb/article-lookup/doi/10.1242/jeb.251313#supplementary-data

## References

**Grosse, I. R., Dumont, E. R., Coletta, C. and Tolleson, A.** (2007). Techniques for modeling muscle-induced forces in finite element models of skeletal structures. *Anat. Rec.* **290**, 1069-1088. doi:10.1002/ar.20568

**Kreyzig, E.** (1979). *Advanced Engineering Mathematics*. 7th edn. Wiley.

**Marcé Nogué, J., De Esteban-Trivigno, S., Escrig, C. and Gil, L.** (2016). Accounting for differences in element size and homogeneity when comparing finite element models: armadillos as a case study. *Palaeontol. Electron.* **19**, 1-22. doi:10.26879/609

**Marcé-Nogué, J., De Esteban-Trivigno, S., Pü schel, T. A. and Fortuny, J.** (2017). The intervals method: a new approach to analyse finite element outputs using multivariate statistics. *Peer J* **5**, e3793. doi:10.7717/peerj.3793

**Strait, D. S., Grosse, I. R., Dechow, P. C., Smith, A. L., Wang, Q., Weber, G. W., Neubauer, S., Slice, D. E., Chalk, J., Richmond, B. G. et al.** (2010). The structural rigidity of the cranium of Australopithecus africanus: implications for diet, dietary adaptations, and the allometry of feeding iomechanics. *Anat. Rec.* **293**, 583-593. doi:10.1002/ar.21122

**Walmsley, C. W., Smits, P. D., Quayle, M. R., McCurry, M. R., Richards, H. S., Oldfield, C. C., Wroe, S., Clausen, P. D. and McHenry, C. R.** (2013). Why the long face? The mechanics of mandibular symphysis proportions in crocodiles. *PLoS ONE* **8**, e53873. doi:10.1371/journal.pone.0053873

Journal of Experimental Biology • Supplementary information
